# Lycopene antagonizes lead toxicity by reducing mitochondrial oxidative damage and mitochondria‐mediated apoptosis in cultured hippocampal neurons

**DOI:** 10.1002/mco2.17

**Published:** 2020-08-27

**Authors:** Mingyue Qu, Yanli Ni, Baoshi Guo, Xin Feng, Zheng Jiang

**Affiliations:** ^1^ The PLA Rocket Force Characteristic Medical Center Beijing China

**Keywords:** hippocampal neuron, lead, lycopene, mitochondria, oxidative stress

## Abstract

Lead (Pb) exhibits serious adverse effects on the central nervous system, and the major pathogenic mechanism of Pb toxicity is oxidative stress. As one of the carotenoid family members with potent antioxidant properties, lycopene has shown its protections by inhibiting oxidative stress damage in numerous models of neurotoxicity. The current study was designed to explore the possible protective property in primary cultured rat hippocampal neurons challenged with Pb. We observed that 5 μM lycopene pretreatment for 4 h efficiently ameliorated Pb‐caused damage in cell viability, accumulation of reactive oxygen species (ROS), and apoptosis in a dose‐dependent manner. Moreover, lycopene (5 μM) attenuated the 50 μM Pb‐induced mitochondrial ROS production, improved the activities of mitochondrial respiratory chain enzymes and ATP production, and ameliorated the 50 μM Pb‐induced depolarization of mitochondrial membrane potential as well as opening of mitochondrial permeability transition pores. In addition, 5 μM lycopene restored the imbalance of Bax/Bcl‐2, inhibited translocation of cytochrome c, and reduced caspase‐3 activation. Taken together, these findings indicate that lycopene antagonizes against Pb‐induced neurotoxicity and the underlying mechanism probably involves reduction of mitochondrial oxidative damage and mitochondria‐mediated apoptosis.

## Graphical abstract

In this study, we demonstrated the neuroprotective effects of lycopene in primary cultured rat hippocampal neurons challenged with lead. The protective effects of lycopene may be attributable to its roles in reducing mitochondrial oxidative damage and mitochondria‐mediated apoptosis.

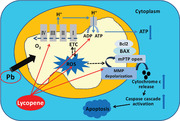

## 1 INTRODUCTION

Lead (Pb) is a ubiquitous toxic environmental pollutant which can be detected in many different biological systems. Pb toxicity remains a major public health concern for the adverse health effects.^1^ Many organ systems are targets for the Pb toxicity, and nervous system seems to be extremely fragile to it. Multiple lines of evidence have indicated that Pb exposure exerts serious damages in the nervous system, including vision and hearing deficits, learning and cognitive impairment, and behavioral abnormalities.^2^ Several studies have shown that Pb is likely to selectively gather in the hippocampus, leading to impairments to hippocampal neurons.^3^ So far, the exact molecular mechanisms are not yet completely clarified. A common perception is that oxidative stress and neuronal apoptosis by mitochondrial dysfunction contribute to the pathogenesis of Pb neurotoxicity.^4^ Multiple previous studies have reported that Pb exposure evokes oxidative stress through massive production of reactive oxygen species (ROS) and antioxidant defense system perturbation.^5^ Mitochondria are the main site of ROS production and appear to have a critical role in the intrinsic pathway of apoptosis. Pb is able to enter into mitochondria and damage the organelle, causing mitochondrial dysfunction and excessive ROS generation.^6^ The elevated ROS levels and mitochondrial abnormalities may trigger a cascade of mitochondria‐mediated apoptosis that contribute to the Pb‐induced neuronal damage and death.^7^

**FIGURE 1:**
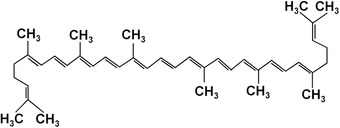
The chemical structure of lycopene

Considering the involvement of oxidative stress in Pb‐induced neurotoxicity, it is plausible to speculate that antioxidants administration may be particularly promising in protection against its toxicity. Lycopene is a natural pigment which is rich in red fruits and vegetables, such as tomatoes, red carrots, pink grapefruits, watermelon, strawberry, and apricots. The potential health benefits of lycopene were reviewed by Caseiro and Ascenso.^8^ As a biologically occurring carotenoid, lycopene exhibits multiple physiological and biochemical functions, such as induction of gap‐junctions communications,^9^ modulation of detoxifying enzymes,^10^ anti‐inflammatory activity,^11^ antiproliferative property,^12^ and immunomodulatory effect.^13^ The health effects of lycopene mainly based on its powerful antioxidant property due to its highly efficient singlet‐oxygen quenching capacity. Lycopene has a structure with 11 linear conjugated double bonds (Figure 1), which makes its potent ability in quenching singlet oxygen twice higher than β‐carotene and 10 times as high as α‐tocopherol.^14^ Numerous studies have proved that lycopene alleviates ROS production and mitigates oxidative stress damage in different experimental models.^15^, ^16^, ^17^ As a natural organic lipid‐soluble pigment, lycopene is able to effectively cross through the blood‐brain barrier, implying that lycopene may play a beneficial role against oxidative impairments in the central nervous system. Indeed, epidemiologic research highlights a statistically inverse relationship between the lycopene concentration in serum and risk of stroke.^18^ It is also found that long‐term intake of lycopene‐rich food is able to lower the risk of cognitive dysfunction^19^ and decrease the mortality of Alzheimer's disease patients.^20^ In laboratory experimental studies, lycopene was observed to protect against the learning‐memory ability impairment of vascular dementia rats by inhibiting oxidative stress in the hippocampus.^21^ Dietary lycopene supplementation was found to attenuate oxidative stress and improve cognitive ability through the Nrf2/NF‐κB pathway in mouse models.^22^, ^23^, ^24^ Lycopene also protects against brain oxidative damage induced by hyperlipidemia in rat.^25^ Moreover, several recent studies have reported the beneficial effects of lycopene on apoptosis mediated by ROS accumulation and mitochondria dysfunction in the 1‐methyl‐4‐phenyl‐1,2,3,6‐tetrahydropyridine or rotenone model of Parkinson's disease,^26^, ^27^ amyloid β model of Alzheimer's disease,^11^, ^28^ and 3‐nitropropionic acid model of Huntington's disease,^29^, ^30^ indicating the potential of lycopene being an efficient agent for treating neurologic lesions associated with oxidative stress. In addition, lycopene exhibits neuroprotections against oxidative damage and apoptosis against tert‐butyl hydroperoxide‐mimicked oxidative stress and oxidative neurotoxicity through the PI3K/Akt pathway.^31^ Previous in vitro studies also showed the remarkable protective effects of lycopene against heavy metal toxicity, such as trimethyltin,^32^ methylmercury^33^ in primary cultured neurons. Collectively, these results imply that lycopene has a wide range of protective activities in the nervous system. Therefore, our study aims to probe the potential effects of lycopene on Pb‐induced neurotoxicity and possible mechanisms for the observed effects.

## 2 RESULTS

### 2.1 Lycopene attenuated oxidative damage induced by Pb

**FIGURE 2:**
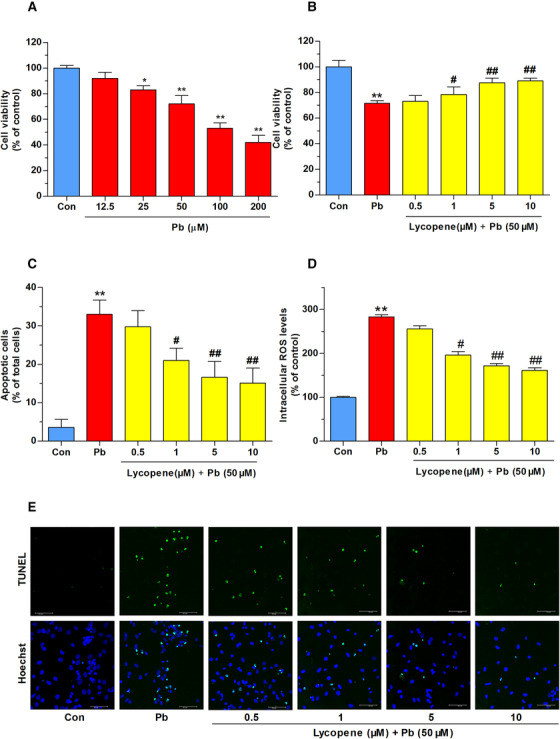
Effects of lycopene on Pb‐evoked oxidative damage. (A) Effects of Pb on neuronal cell viability. CCK‐8 assay was used for measuring the cell viability. Neurons exposure with Pb (12.5‐200 μM) for 24 h caused a concentration‐dependent decrease in the cell viability. Effects of lycopene on Pb‐caused (B) cell viability loss, (C) apoptosis, and (D) oxidative stress. Neurons were treated with Pb (50 μM) for 24 h in the presence or absence of lycopene pretreatment (0.5, 1, 5, and 10 μM) for 4 h. The neurons were analyzed for (B) cell viability, (C) apoptosis, and (D) intracellular ROS levels. The values of cell viability in the control group were set at 100%, and results were expressed as a percentage of the control. (C) TUNEL staining was used for the detection of neuronal apoptosis. Neuronal apoptosis rates were calculated as the ratio of the numbers of TUNEL‐positive neurons to the number of total neurons. (E) Representative TUNEL staining images were shown. (D) DCFH‐DA staining was used for measuring the intracellular ROS levels. Results of ROS levels were expressed as a percentage of the control (as 100%). Values are shown as mean ± SD, *P < .05, **P < .01 vs control, #*P* < .05, ##*P* < .01 vs Pb‐treated group, *n* = 4.

Hippocampus is a major target of Pb exposure and primary cultured rat hippocampus neurons can replicate some normal physiological and pathological characteristics of experimental animals, and it is easy to control experimental conditions. Therefore, we select primary cultured rat hippocampus neurons to identify the lycopene on Pb‐induced toxicity. First of all, for selecting a working dose for subsequent studies, neurons were treated with ascending concentrations of Pb (From 12.5 to 200 μM) for 24 h. The outcome of CCK‐8 detection exhibited that the viability of neurons was dramatically reduced by Pb treatment at 25 (*P*  <   .05), 50, 100, and 200 μM (*P*  <   .01), as illustrated in Figure 2A. A moderate toxic effect was presented at the concentration of 50 μM. Thus, 50 μM Pb was selected to stimulate neurons in the following experiments.

To probe the effects of lycopene on Pb‐induced toxicity, we used lycopene at four gradient does (0.5, 1, 5, or 10 μM) to treat neurons before Pb (50 μM) exposure. We selected this dose range of lycopene according to our preliminary experiments and previous study that over 10 μM of lycopene exhibited toxic effect on cell viability of cultured neurons. Results in Figure 2B, C, and E revealed that lycopene pretreatment significantly inhibited both the cell viability loss and neuronal apoptosis induced by Pb exposure in a dose‐dependent manner. Excessive ROS plays a key role in the oxidative stress damage induced by Pb. Intracellular levels of ROS were measured using dichlorofluorescein diacetate (DCFH‐DA) staining. As described in Figure 2D, intracellular ROS level in neurons treated with 50 μM Pb for 24 h was largely higher than control, and lycopene mitigated this high level of ROS in a dose‐dependent manner. Interestingly though, there were almost similar effects of 5 and 10 μM lycopene on neuronal damage and ROS accumulation. According to our previous studies,^32^, ^33^ no toxic effect of lycopene up to a dose of 10 μM was found in neuronal cells. The protective effect of 5 to 10 μM of lycopene may have reached its plateau levels. This range may be the maximum protective effect dose of lycopene to balance its own toxicity. Therefore, lycopene at the dose of 5 μM was selected for exploring the mechanism underlying its neuroprotection in the subsequent study.

### 2.2 Lycopene attenuated mitochondrial oxidative stress and dysfunction induced by Pb

**FIGURE 3:**
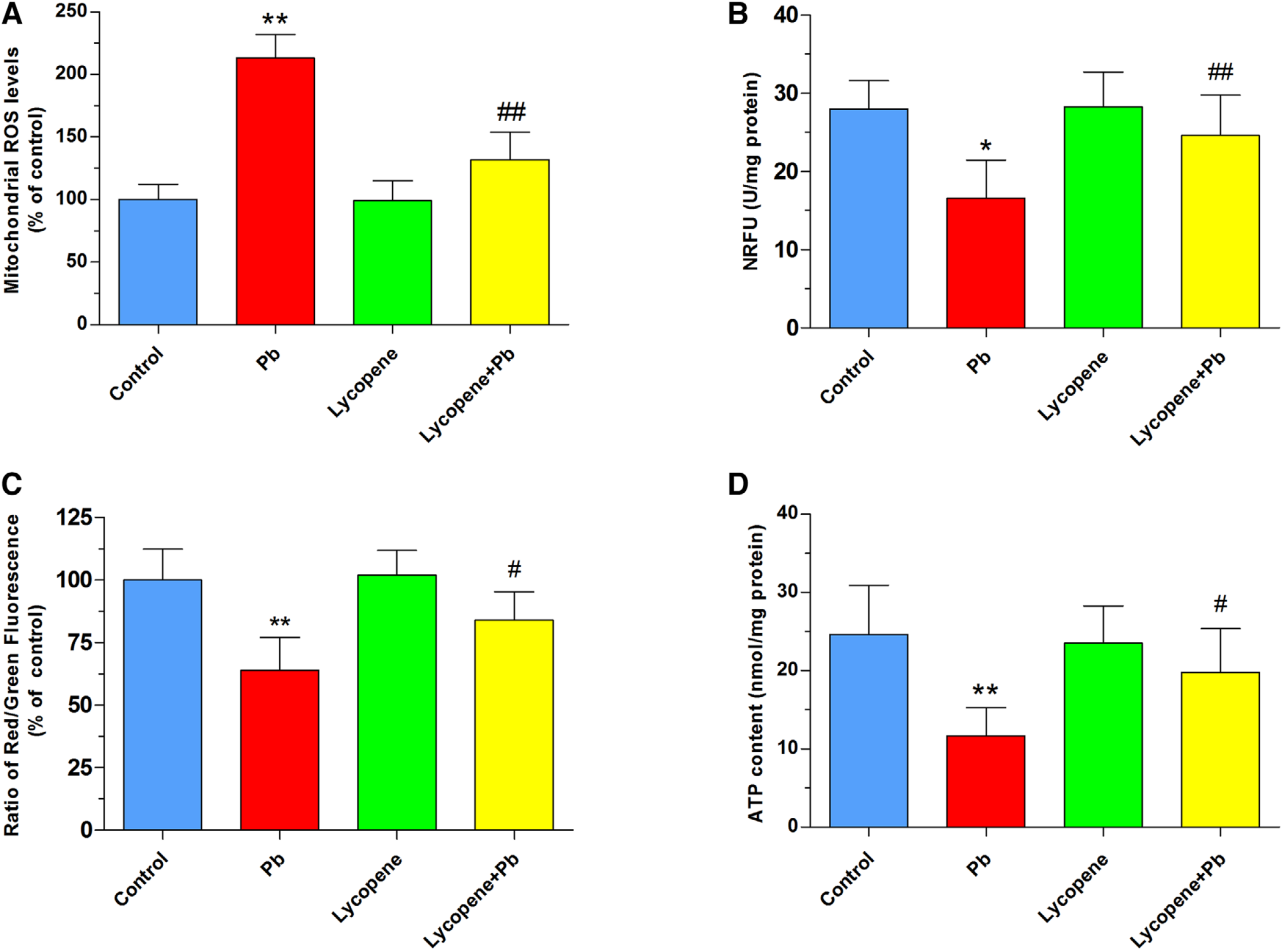
Effects of lycopene on Pb‐evoked mitochondrial oxidative stress and dysfunction. The cultured neurons were exposed for 24 h to 50 μM Pb with or without a 4‐h lycopene preincubation. (A) The mitochondrial ROS level was detected using MitoSOX Red. Results were presented as percentages of control (as 100%). (B) The opening of mPTP was determined using an mPTP assay kit. The calcein fluorescence represented the level of mPTP opening. The values of calcein fluorescence were quantified and expressed as NRFU (U/mg protein). (C) The level of MMP was determined using JC‐1 staining. Data were presented as a percentage of control (as 100%). (D) The intracellular ATP level was detected using an ATP determination kit and normalized to cell protein concentration. ATP data were presented as nmol/mg protein. *P < .05, **P < .01 vs control, #*P* < .05, ##*P* < .01 vs Pb‐treated group. Values are mean ± SD, *n* = 4

Mitochondria are regarded as one of the main sources of ROS production and one of the main targets of ROS. For these reasons, we investigated the role of mitochondria in producing ROS by assessing mitochondrial ROS levels with MitoSOX Red dye. As illustrated in Figure 3A, after 24 h exposure to 50 μM Pb, the cultured neurons displayed significantly higher mitochondrial ROS level compared with control (*P* < .01). When the neurons preincubated with 5 μM lycopene, Pb‐induced mitochondrial ROS overproduction was significantly prevented (*P* < .01).

Results in Figure 3B illustrated that mitochondrial calcein fluorescence in neurons exposed to 50 μM Pb for 24 h was found to be markedly decreased as compared to control (*P* < .05), representing the abrupt opening of mitochondrial permeability transition pores (mPTP). However, lycopene mitigated the Pb‐caused mPTP opening significantly (*P* < .01).

Impaired mitochondrial membrane potential (MMP) is an early universal event of apoptosis and a sensitive indicator of mitochondrial dysfunction. Treatment with 50 μM Pb induced a marked reduction in the ratio of red/green fluorescence (*P* < .01), indicating MMP depolarization in hippocampal neurons. In contrast, the MMP dissipation was largely restored when neurons were pretreated with 5 μM lycopene as described in Figure 3C (*P* < .05).

The intracellular ATP concentration is a critical parameter for mitochondrial dysfunction. Results in Figure 3D indicated that Pb exposure significantly decreased ATP contents (*P* < .01), while lycopene treatment alone had no effect on intracellular ATP levels. However, the impaired production of ATP was able to be efficiently restored by lycopene preincubation (*P* < .05).

### 2.3 Lycopene attenuated mitochondrial respiratory complex anomalies induced by Pb

**FIGURE 4:**
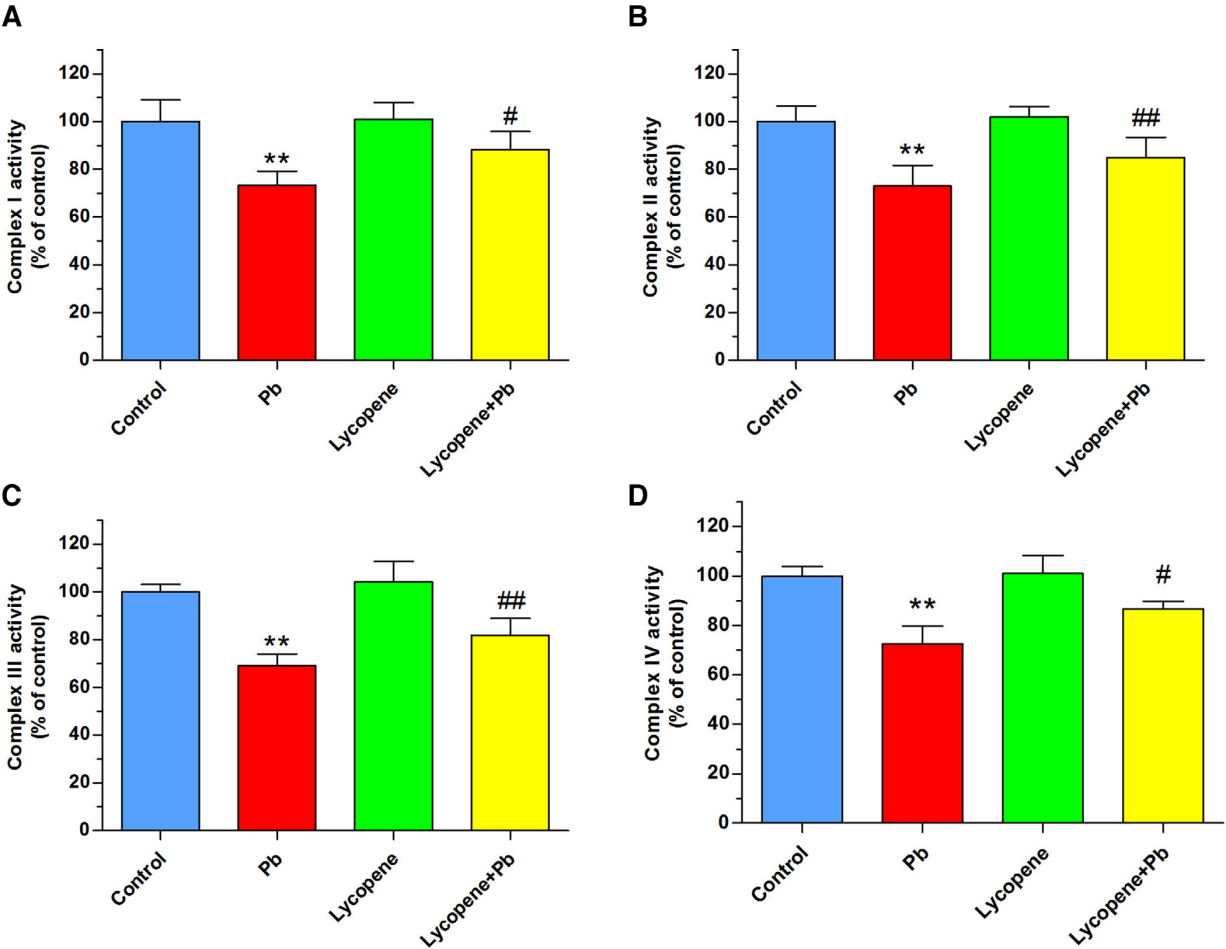
Effects of lycopene on Pb‐induced impairment of mitochondrial respiration. The cultured neurons were exposed for 24 h to 50 μM Pb with or without a 4‐h lycopene preincubation. Enzymatic activities of complex I (A), complex II (B), complex III (C), and complex IV (D) of the mitochondrial respiratory chain in mitochondria isolated from neurons were determined. Results were expressed as a percentage of the control (as 100%). **P < .01 vs control, #*P* < .05, ##*P* < .01 vs Pb‐treated group. Values are mean ± SD, *n* = 4

Mitochondrial oxidative stress has been known as the result of respiratory complex dysfunction. Therefore, we assayed mitochondrial respiratory chain complexes activities in isolated mitochondria. Results in Figure 4 indicated that the mitochondrial respiratory complexes activities were remarkably suppressed in neurons exposed Pb (50 μM) (*P* < .01). In contrast, those suppressions were notably attenuated by lycopene preincubation (*P* < .05, *P* < .01).

### 2.4 Lycopene reversed the ratio of Bax/Bcl‐2 expression in Pb‐treated neurons

**FIGURE 5:**
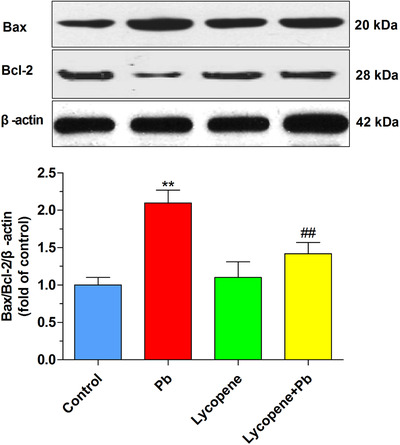
Effects of lycopene on the Pb‐induced alteration of the ratio of Bax/Bcl‐2 expression. The cultured neurons were exposed for 24 h to 50 μM Pb with or without a 4‐h lycopene preincubation. Western blot was performed for assessing Bax and Bcl‐2 expression, and the representative images of immunoblots were shown. β‐actin was used as a loading control. Quantitative analysis of the ratio of Bax/Bcl‐2 was presented as fold of control. **P < .01 vs control, ##*P* < .01 vs Pb‐treated group. Values are mean ± SD, *n* = 4

To probe the role of Bcl‐2 family in the protective effects of lycopene on Pb toxicity, we determined the Bcl‐2 and Bax expression. Compared with the control group, neurons exposed to 50 μM Pb for 24 h exhibited an upregulation of Bax expression whereas a downregulation of Bcl‐2 expression. As a result, statistical analysis showed ratio of Bax/Bcl‐2 raised significantly (*P* < .01). In contrast, such imbalance of Bax/Bcl‐2 was considerably restored by lycopene preincubation (*P* < .01), highlighting its strong antiapoptosis function (Figure 5).

### 2.5 Lycopene prevented the Pb‐caused cytochrome c redistribution and caspase‐3

Cytochrome c redistribution from mitochondria to cytosol and consequent caspase‐3 activation are two early events of the mitochondrial apoptosis pathway. As illustrated in Figure wwww6A, Pb exposure raised cytosolic cytochrome c level, accompanied by a reduction in cytochrome c levels in mitochondrial faction, indicating the translocation of cytochrome c (*P* < .01). However, the Pb‐caused cytochrome c release was efficiently attenuated by lycopene preincubation (*P* < .01). Caspase‐3 can be potently activated by cytochrome c redistribution. As described in Figure 5B, the expression of 17 kDa active subunit of caspase‐3 in Pb‐treated neurons was largely upregulated (*P* < .01), which was obviously ameliorated by lycopene preincubation (*P* < .05).

**FIGURE 6:**
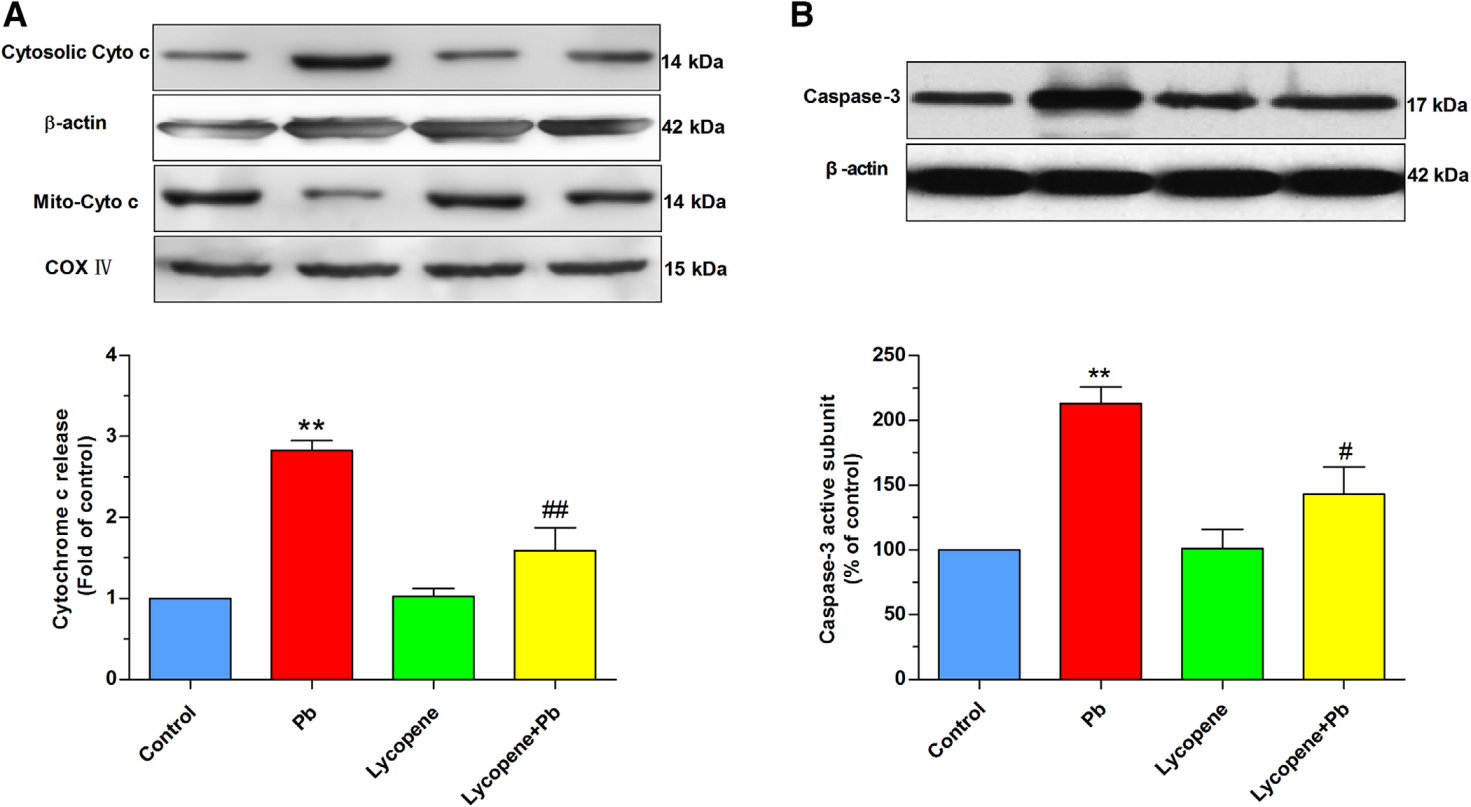
Effects of lycopene on the Pb‐evoked redistribution of cytochrome c and activation of caspase‐3. The cultured neurons were exposed for 24 h to 50 μM Pb with or without a 4‐h lycopene preincubation. (A) Cytochrome c redistribution from mitochondria to cytosol was evaluated using Western blot analysis. Cytochrome c oxidase subunit IV (COX IV) was used as a mitochondrial loading control. β‐actin was used as loading control for cytosolic fraction. Results were calculated as the ratio of cytosolic to mitochondrial cytochrome c levels and expressed as fold of control. (B) Caspase‐3 activation was investigated by detecting the expression of the 17 kDa active subunit of caspase‐3. The loading control was β‐actin. Data were expressed as a percentage of control (as 100%). **P < .01 vs control, #*P* < .05, ##*P* < .01 vs Pb‐treated group. Values are mean ± SD, *n* = 4

## 3 DISCUSSION

In the current study, we uncovered the role of lycopene in antagonizing Pb‐induced neurotoxicity in a cellular model of primary cultured rat hippocampal neurons. We observed that preincubation of lycopene significantly attenuated Pb‐induced oxidative damage, as evidenced by the promoted cell viability, the reduced intracellular ROS level, and neuronal apoptosis. Furthermore, lycopene ameliorated the mitochondria‐derived ROS production, suppressed the opening of mPTP and collapse of MMP, and improved the mitochondrial respiratory complexes activities and restored the generation of ATP in neurons exposed to Pb. Moreover, lycopene alleviated the Pb‐induced increase in the ratio of Bax/Bcl‐2, redistribution of cytochrome c, and the consequent caspase‐3 activation. The neuroprotective mechanism of lycopene against Pb toxicity may be associated with reducing mitochondrial oxidative damage and mitochondria‐mediated apoptosis.

Special chemical structure of lycopene makes it a highly efficient scavenger toward superoxide anions, hydroxyl radicals, and singlet oxygens.^34^ Excessive generation of ROS seems to be one underlying mechanism in Pb toxicity. ROS can impair many cellular components, such as DNA, protein, and lipid membranes, eventually leading to apoptosis. Several previous studies have demonstrated that lycopene interacts with ROS and thereby prevents ROS‐induced oxidative damage. It is shown that lycopene improved the expression of antioxidant enzymes and reduced ROS production through the PI3K‐Akt pathway in human mesenchymal stem cells.^35^ It is also reported that lycopene suppress apoptosis by decreasing cellular ROS, prohibiting activation of NF‐κB in neuronal cells.^36^ In a rabbit restenosis model, lycopene was found to have effect on regulation of blood lipid and inhibited the oxidative damage.^37^ The results of our study confirmed previous findings, demonstrating that lycopene attenuated the Pb‐caused neuronal injury and ROS overproduction in a dose‐dependent manner. Consistent with these findings, similar protection of lycopene against neuronal oxidative damage is reported both in a tert‐butyl hydroperoxide‐treated cell model^31^ and in a vascular dementia animal model.^21^

Exposure of Pb can cause in a wide range of impairment in organs including heart, liver, and kidneys and systems including the reproductive system, central and peripheral nervous systems, and endocrine system. Several lines of evidence reveal a critical role for the mitochondria in regulating apoptosis. It is reported that Pb tends to localize in the mitochondria and impair mitochondrial function, causing excessive mitochondrial ROS generation. The accumulated ROS could injure mitochondrial function deeply and aggravate ROS generation, trigger a vicious cycle that finally leads to mitochondrial dysfunction and neuronal death.^38^ Our results revealed that Pb exposure caused a marked increase in mitochondria‐derived ROS generation, and this burst of ROS formation was significantly decreased by lycopene. Although the free radical quenching activity of lycopene likely contributes to its antiapoptotic effect, other modes of direct action on mitochondria have also been suggested. Previous studies revealed the beneficial effect of lycopene on mitochondria in neurons challenged by amyloid β^39^ and in cardiomyocytes following phenylephrine treatment.^40^ For this reason, mitochondrial function‐related parameters were further investigated.

Mitochondrial permeability transition is a representative event and a central coordinator of apoptosis. One of the critical consequences of the mitochondrial permeability transition is the release of apoptosis‐promoting factors which results in apoptosis. The opening of mPTP and collapse of MMP are two important characterizations of the mitochondrial permeability transition.^41^, ^42^ It is reported that Pb exposure can evoke the mPTP opening and depolarization of membrane potential.^43^ Consistent with previous studies, we found that Pb treatment induced opening of mPTP, loss of MMP, and decrease in ATP content, indicating significant impairment in mitochondrial function. In contrast, lycopene prominently alleviated the Pb‐induced mitochondrial dysfunction. MMP is essential for driving the production of ATP. Lycopene boosts ATP production, maybe by maintaining MMP. This point is supported by a previous report that lycopene prevented MMP(+)‐evoked depolarization of MMP and reduction in ATP production in SH‐SY5Y cells.^44^ Alternatively, there may be another explanation for the observed restoration of mitochondrial energetic efficiency. Lycopene may also improve ATP production by improving enzymatic activities of complexes in the mitochondrial respiratory chain. As observed in our study that lycopene pretreatment ameliorated the impairment of activities of respiratory enzymes in neurons exposed to Pb. Our findings are in conformity with the previous reports that lycopene enhances the activities of mitochondrial respiratory chain in rat model treated with Aβ^45^ or colchicine.^46^

Bcl‐2 family plays a pivotal role in regulation of the permeability of mitochondrial membrane and initiation of mitochondria‐mediated apoptosis. When the expression of Bax increased, this apoptosis‐promoting protein can oligomerize in the outer mitochondrial membrane, resulting in transition of mitochondrial membrane permeability and consequently apoptogenic factors release. However, the Bax oligomerization can be disrupted by heterodimerization with the Bcl‐2, thereby stabilizing mitochondrial membrane permeability. Thus, the ratio of Bax/Bcl‐2 determines the trend of apoptosis. Pb‐induced imbalance of the Bax/Bcl‐2 ratio has been revealed in several experimental models.^47^, ^48^ Bcl‐2 reduction and Bax elevation induced by Pb exposure were observed in our study. However, this raised ratio of Bax to Bcl‐2 level was reversed by lycopene, indicating that lycopene may antagonize Pb neurotoxicity through regulating the balance between these molecules. Our data collaborated with the results of previous studies that lycopene modulated the balance of Bax/Bcl‐2 expression and inhibits neuronal apoptosis in ischemia/reperfusion‐treated gerbils^49^ and monosodium glutamate (E621)‐treated rats.^50^ The imbalance of Bax/Bcl‐2 might result in mitochondrial dysfunction and activating the downstream caspase pathway. Here, we found that the cytochrome c redistribution to cytosol and caspase‐3 activation was abolished by lycopene, suggesting that lycopene blocked the mitochondria‐mediated apoptosis pathways. These findings are in line with previous observation in neurotoxicity models established by exposure of cultured neurons to trimethyltin^32^ or tert‐butyl hydroperoxide.^31^

**FIGURE 7:**
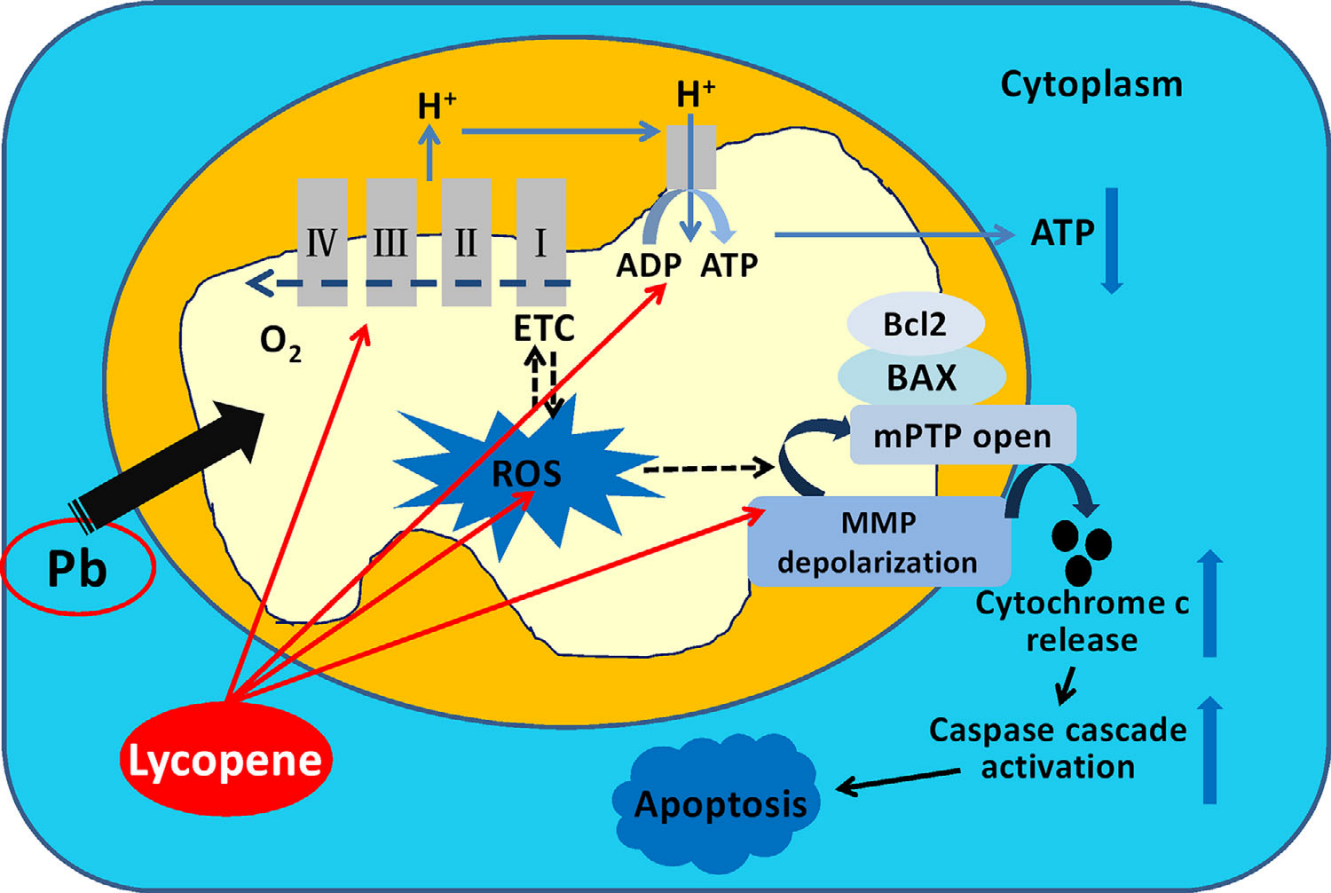
Proposed mechanism by which lycopene protects against Pb toxicity by reducing mitochondrial oxidative damage and mitochondria‐mediated apoptosis in cultured hippocampal neurons. Lycopene scavenges Pb‐induced excessive ROS production by inhibiting MMP depolarization and mitochondrial membrane permeability transition pore opening. Moreover, lycopene also restores mitochondrial respiratory chain function and ATP generation and attenuates Pb‐induced alteration of the ratio of Bax/Bcl‐2 expression as well as release of cytochrome c and activation of caspase‐3. Upregulation and downregulation are represented by upward (↑) and downward (↓) arrows in blue color

Based on the above results, we may conclude that the beneficial effect attributed to lycopene might be mainly conducted by alleviating mitochondrial oxidative damage and mitochondria‐mediated apoptosis. Among several known physiological and biochemical functions of lycopene, the exceptional antioxidant properties of lycopene may be largely responsible for the observed neuroprotection. Besides the direct effects of lycopene on oxidative stress by its free radical scavenging ability, lycopene may also act directly on mitochondria due to the central role of mitochondria in ROS generation. Lycopene may suppress the Pb‐induced oxidative stress by maintaining mitochondrial function, as evidenced by improved mitochondrial respiratory complexes activities and inhibited mitochondrial permeability transition. On the other hand, lycopene may also regulate Bcl‐2 family proteins to maintain mitochondrial membrane integrity and inhibit proapoptotic factors release, therefore blocking the mitochondria‐mediated apoptosis pathways. Therefore, we believe that the mitochondrial mechanism probably plays a predominant part in the neuroprotection of lycopene (Figure 7).

In summary, lycopene could antagonize Pb toxicity in a cellular model with primary cultured rat hippocampal neurons, which provides the evidence for this agent as a promising therapeutic agent for Pb toxicity. The mechanism of such beneficial effects may be related to reducing mitochondrial oxidative damage and subsequent mitochondria‐mediated apoptosis. However, the current study was only at in vitro experiment level, further in vivo researches and the related signal pathways are required to clarify this issue.

## 4 MATERIALS AND METHODS

### 4.1 Neuronal culture and treatment

Hippocampal neurons were cultured follow the method that detailed in our previous study.^32^ Experiments were performed complying with the guideline of experimental animal use of the PLA Rocket Force Characteristic Medical Center. Briefly, under a dissecting microscope, hippocampi were separated from newborn Sprague‐Dawley rats and hippocampal tissue was trypsinized (0.05% for 10 min at 37°C; Gibco) and mechanically dispersed. Then, dispersed cells were plated at a density of 5 × 10^5^ cells/mL on poly‐l‐lysine‐coated culture plates in DMEM/F‐12 medium, supplemented with 10% fetal bovine serum and 10% horse serum, and 50 mg/mL penicillin/streptomycin (Sigma‐Aldrich) for 24 h. Afterwards, the culture medium was replaced with Neurobasal medium containing 2% B27 supplement containing 0.5 mM l‐glutamine (Gibco). Thereafter, half of the culture medium was removed and fresh medium was added every 2 days. Cultured neurons were incubated in an incubator with 5% CO_2_ and 95% O_2_ at 37°C and used for cellular experiments at day 8.

Lead acetate and lycopene were purchased from Sigma‐Aldrich. Lycopene is unstable under the condition of light, heat, and oxygen. To prevent isomerization and oxidation, lycopene was dissolved in tetrahydrofuran containing 0.025% butylated hydroxytoluene. The exposure solutions were freshly prepared before use in the culture medium and sterilized by filtration. Neurons were incubated with lycopene for 4 h, then expose to lead acetate for 24 h. Doses used in this study were according to previous studies on the protection of lycopene in oxidative damage models and Pb toxicity models.^32^, ^51^

### 4.2 Cell viability assays

Cell Counting Kit‐8 (CCK‐8) was used for cell viability measurement following the manufacturer's procedure (Dojindo, Japan). Briefly, neurons seeded in the 96‐well plates were mixed with 10 μL CCK‐8 solutions at 37°C. After 2 h incubation, the absorbance at 450 nm was determined using a microplate reader. Results were normalized to the control group using the percentage value. The experiment was repeated four times.

### 4.3 TUNEL staining

Terminal deoxynucleotidyltransferase‐mediated dUTP nick end labeling (TUNEL) staining for neuronal apoptosis was conducted using the In Situ Cell Death Detection POD Kit (Roche, USA). Culture media was removed after treatments. Neurons seeded on poly‐l‐lysine‐coated glass coverslips were washed twice with warmed phosphate‐buffered saline (PBS). Then 4% paraformaldehyde PBS solution was added to fix the neurons at room temperature for 20 min. 0.1% Triton X‐100 in 0.1% sodium citrate was then used to permeabilize the cells for 2 min on ice. Then the cells were washed four times with PBS and mixed with 50 μL of TUNEL reaction mixture and maintained at 37°C for 60 min. Hoechst 33258 was used to stain the nuclei. And stained cells were observed by an inverted fluorescence microscope. For each coverslip, six random fields were selected, and 100 cells (Hoechst‐stained) were counted in every field. Results were expressed as the ratio of apoptotic neurons to total neurons of four independent experiments.

### 4.4 Assays for intracellular ROS

A fluorometric method was used to detect intracellular ROS level employing the fluorescent probe DCFH‐DA. Neurons were seeded into 96‐well plates with 1 × 10^4^ cells per well. Neurons were rinsed twice with PBS, then 10 μM DCFH‐DA was added to the culture and maintained for 20 min at 37°C under dark conditions. After incubation, fluorescence intensities were recorded using a microplate reader at 488 nm for excitation and at 525 nm for emission. ROS levels were expressed as percentage of control.

### 4.5 Assays for mitochondrial ROS

MitoSOX Red (Invitrogen) was employed to measure mitochondrial level of ROS following the producer's instruction. In short, neurons were plated into 96‐well plates with 1 × 10^4^ cells per well. After the designated treatments, 5 μM MitoSOX Red was mixed with culture medium and maintained with neurons for 10 min at 37°C under dark conditions. Afterwards, the fluorescence intensity was determined with excitation at 510 nm and emission at 580 nm using a microplate reader. Results were expressed as percentage of control.

### 4.6 Measurement of mPTP opening

The mPTP opening was detected by using an mPTP assay kit (Genmed Scientifics) based on the calcein‐AM/cobalt method following the manufacturer's instructions. Neurons were plated in 24‐well plates (5 × 10^4^ cells per well). After the designated treatments, neurons were rinsed twice with PBS and the staining was done by incubating with calcein‐AM (1 μM per well) and cobalt dichloride (1 mM per well) for 20 min at 37 °C. The intensities of fluorescence were assessed at 488 nm for excitation and at 505 nm for emission. The mPTP opening of each experimental group was represented by normalized fluorescence to protein concentration.

### 4.7 Detection of mitochondrial membrane potential

JC‐1 probe was applied for measurement of MMP as per the manufacturer's protocol (Beyotime, China). Briefly, after the designated treatments, neurons were incubated with JC‐1 for 20 min at 37°C under dark conditions. The intensities of green and red fluorescence were detected by the microplate reader at 514/529 nm and 585/590 nm for excitation/emission. The MMP levels were counted as the ratio of red fluorescence to green fluorescence and represented as percentages of control.

### 4.8 Determinations of cellular ATP levels

ATP content was detected by the luciferin‐luciferase reaction with an ATP Determination Kit (Beyotime). In short, neurons were plated into 6‐well plates with 1 × 10^6^ cells per well. After the designated treatments, the neurons were collected and lysed with 0.2 mL lysis reagent. Supernatant was collected by centrifuging the lysate at 12 000 rpm for 10 min and mixed with 100 μL ATP testing solution, and the reading for the mixture was immediately recorded on a microplate reader. ATP content was calculated by a standard curve and normalized to the protein content of the neurons.

### 4.9 Mitochondrial respiratory chain complexes activity detection

The Mitochondria Isolation Kit (Pierce) was used to isolate mitochondria from the cultured neurons as per the manufacturer's protocol. Purified mitochondria from every group were applied to detect the activities of mitochondrial respiratory complexes I, II, III, and IV through the colorimetric method as described before.^39^ Activities of the mitochondrial complex were normalized to the protein concentration, and results were represented as percentages of control. All of these experiments were repeated four times.

### 4.10 Western blotting

Neurons were collected and lysed in RIPA buffer to extract the whole cell protein. The total cell fractions were used to detect Bcl‐2, Bax, and caspase‐3 protein expressions while the mitochondrial and cytosolic fractions, separated and prepared using the Mitochondria Isolation Kit, were used to assessing cytochrome c. Protein contents were measured with a BCA Kit (Beyotime). Western blot analysis was performed using an Odyssey infrared scanner as described in our previous study.^39^ Equal quantity of proteins was loaded on sodium dodecyl sulfate (SDS) polyacrylamide gel. After electrophoresis, SDS polyacrylamide gel was transferred to nitrocellulose membranes and probed with primary antibodies as indicated: rabbit anti‐Bcl‐2, rabbit anti‐Bax, rabbit anticytochrome c (1:1000; Cell Signaling Technology); mouse anti‐caspase‐3 (1:2000; Abcam); mouse anti‐β‐actin and mouse anti‐COX IV monoclonal (1:500; Sigma‐Aldrich). The following secondary antibodies were used: donkey anti‐rabbit and donkey anti‐mouse (LI‐COR). The fluorescent of each band was calculated by the Odyssey infrared imaging system and normalized to loading control (β‐actin or COX IV).

### 4.11 Statistical analysis

All the data were represented as the mean ± SD. Statistical comparisons were analyzed by analysis of variance (ANOVA) and Fisher's least‐significant difference post hoc test with GraphPad Prism 5.0 software. Results were reported as significant when *P* < .05.

## AUTHORS’ CONTRIBUTIONS

Mingyue Qu and Xin Feng designed the experiments; Yanli Ni and Zheng Jiang performed the experiments; Mingyue Qu and Baoshi Guo analyzed the experimental data and wrote the manuscript; and all authors have read and approved the final manuscript.
